# Scleral Fixation of Intraocular Lenses: Outcomes From MA60AC Intraocular Lens Fixation With a 10-0 Prolene Suture vs Akreos Intraocular Lens Fixation With a Polytetrafluoroethylene (Gore-Tex) Suture

**DOI:** 10.1177/24741264251412080

**Published:** 2026-02-16

**Authors:** Guilherme Marge de Aquino Guedes, Daniel Lani Louzada, Rodrigo Antonio Brant Fernandes, Arthur Gustavo Fernandes

**Affiliations:** 1 Ophthal Hospital Especializado, São Paulo, SP, Brazil; 2 Department of Visual Sciences and Ophthalmology, Federal University of São Paulo, São Paulo, SP, Brazil; 3 Roski Eye Institute, University of Southern California, Los Angeles, CA, USA; 4 Department of Anthropology, University of Calgary, Calgary, AB, Canada

**Keywords:** complications of anterior segment surgery, IOL dislocations/implantations, surgical techniques and maneuvers, vitreoretinal surgery

## Abstract

**Purpose:** To compare the outcomes of intraocular lens (IOL) scleral fixation using Akreos (Bausch + Lomb) lenses with polytetrafluoroethylene (Gore-Tex CV-8, W.L. Gore & Associates) sutures vs 3-piece IOLs (Alcon MA60AC) with 10-0 Prolene (Ethicon, a Johnson & Johnson Company) sutures. **Methods:** Patients undergoing pars plana vitrectomy between January 2019 and July 2021 were retrospectively selected regardless of the technique used. Patients who completed at least 6 months of follow-up were invited to a new clinic visit to evaluate outcomes of their procedures. The protocol included intraocular pressure assessment using a Goldmann tonometer, subjective refraction, corneal topography with a Pentacam HR (Oculus, Optikgeräte GmbH), IOL decentration assessment on biomicroscopy via slitlamp, and optic disc cupping evaluation by indirect ophthalmoscopy. Complications such as corneal edema, ocular hypertension, hypotony, hyphema, IOL decentration, cystoid macular edema, vitreous hemorrhage, suture breakage, retinal detachment, or uveitis–glaucoma–hyphema syndrome were investigated. **Results:** A total of 20 eyes from 20 participants were classified into 2 groups according to surgical technique: Akreos plus GoreTex (n=11) or MA60AC plus Prolene (n=9). The results showed no statistically significant differences between the 2 groups in terms of refractive outcomes, postoperative complications, or demographic variables, suggesting both techniques provide similar efficacy and safety. **Conclusions:** Both the Akreos with Gore-Tex and MA60AC with Prolene scleral fixation techniques are effective and safe for patients requiring IOL implantation in the absence of adequate capsular support. Further research is needed to solidify these observations and guide future clinical decisions.

## Introduction

Suturing intraocular lenses (IOLs) to the sclera is a proven and effective method for implanting IOLs in aphakic eyes with inadequate capsular support. Various IOL types are in use and range from polymethyl methacrylate lenses (with or without haptic eyelets) to foldable single- or multipiece IOLs that allow for smaller incisions. The number of scleral fixation points typically ranges from 2 to 4, with the aim of minimizing IOL tilt and enhancing lens stability and centration. Common suture materials include polypropylene and polytetrafluoroethylene (Gore-Tex, W.L. Gore & Associates) sutures, though their use for scleral fixation is considered off-label.

Compared with anterior chamber fixation, scleral fixation of posterior chamber IOLs is increasingly used, owing to several advantages. These include greater proximity of the IOL to the nodal point and the center of eye rotation, lower potential for pupil blockage, reduced risk of corneal endothelium loss, and absence of interference with aqueous humor drainage through the iridocorneal angle.^1^ Additionally, pars plana vitrectomy (PPV) combined with scleral fixation is beneficial in cases involving retained crystalline fragments, dislocated IOLs, or posteriorly subluxated lenses.^2,3^

Recently, Gore-Tex sutures, which are monofilament sutures featuring polytetrafluoroethylene biomaterial, have emerged in ophthalmology as a substitute for Prolene (Ethicon, a Johnson & Johnson Company) in IOL fixation. The former has demonstrated resilience in humans even after decades of use,^4^ whereas the latter has exhibited susceptibility to erosion and breakage over time.^5^

The purpose of this study is to compare the outcomes of scleral fixation of IOLs employing PPV performed using Akreos (Bausch + Lomb) lenses with CV-8 Gore-Tex sutures vs 3-piece lenses (MA60AC, Alcon) with 10-0 Prolene sutures.

## Methods

### Study Population

The study included patients who underwent posterior PPV with scleral fixation, owing to the absence of adequate support for the IOL in the capsular bag or ciliary sulcus at the time of surgery. We retrospectively selected patients from Ophthal Hospital Especializado Ltda, a private reference center for ocular surgery in São Paulo, Brazil, who were operated on between January 2019 and July 2021. Patients were chosen regardless of the surgical technique used (decided at the surgeon’s discretion), and all procedures were performed by the same surgeon (R.A.B.F.). Patients who completed at least 6 months of follow-up were invited for a new clinic visit to evaluate the outcomes of their treatments.

The study protocol was approved by the H. Olhos Institutional Review Board and carried out according to the tenets of the Declaration of Helsinki.

### Surgical Techniques

#### PPV With Scleral Fixation of MA60AC Lens and 10-0 Prolene Suture

After positioning the inferior temporal infusion, 2 diametrically opposed points relative to the cornea were selected with the assistance of a toric lens marker. A limbal conjunctival peritomy was made at each junction, with cauterization of the exposed areas. At the markings, a triangular scleral flap was created, with a thickness of one-third of the sclera, sides measuring 3 mm, and the side meeting the corneal limbus maintaining continuity with the sclera.

Approximately 1 mm from the limbus below 1 of the flaps, a 10-0 Prolene suture was trans-fixated into the sclera and passed behind the iris, exiting the eye through an insulin needle positioned in the opposite flap under the same conditions. Through a main incision made with a 2.75-mm blade, the suture was drawn out of the eye and cut in half. Each of the ends was tied to the distal third of the MA60AC lens haptic using the 3-1-1 suture technique.

**Figure 1.**
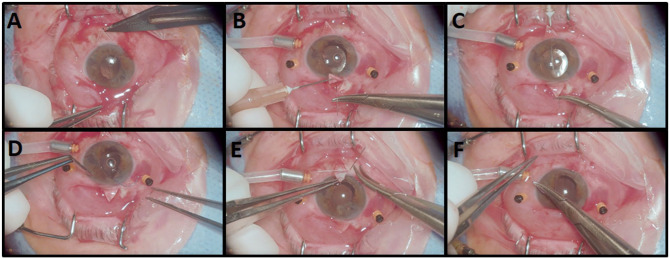
Surgical steps of vitrectomy with scleral fixation of an MA60AC intraocular lens (IOL) using a 10-0 Prolene (Ethicon, a Johnson & Johnson Company) suture. (A) Creation of the scleral flap; (B) initial passage of the Prolene suture; (C) completion of suture passage with the aid of an insulin needle; (D) fixation of the Prolene suture to the distal third of the IOL haptic through the main incision; (E) passage of the suture needle through the sclera after positioning the IOL inside the eye; (F) conjunctival closure using a Vicryl (Ethicon) suture following IOL fixation.

The main incision was enlarged, and the IOL was positioned in the posterior chamber. An accessory incision was created with a 15° blade to assist in intraocular manipulation. The remaining ends of the sutures were pulled and secured to the sclera beneath the flap, ensuring that the lens was well-positioned and the sutures were not exposed. The flaps and the conjunctiva adjacent to them were sutured with 8.0 Vicryl (Ethicon). The accessory and main incisions were hydrated with balanced saline solution, and if leakage occurred, suturing was performed with 10-0 nylon.^1,6,7^ The surgical steps of PPV with scleral fixation of an MA60AC lens and 10-0 Prolene suture are shown in Figure 1.

#### Fixation of the Akreos Lens With CV-8 Gore-Tex Sutures

After positioning the infusion via pars plana in the inferior temporal region, a toric lens marker was used to locate 2 points on the limbus, separated from each other by 180°. Conjunctival peritomy was performed adjacent to these points, followed by hemostasis via cautery of the exposed area. For each previously marked point, a new marking was made 2 mm from the limbus, and from this new marking, 2 new points were made, 2 mm from the limbus and 4 mm apart, totaling 4 points for the sclerotomies. If vitrectomy was performed during fixation, the upper sclerotomies (temporal and nasal) were used to position the 23-gauge trocars.

A main corneal incision was made using a 2.75 mm blade, and another accessory incision was made with a 15° blade, both superiorly, for manipulation in the anterior segment. An 8.0 Gore-Tex suture was equally cut into 2 pieces, and each piece was passed in a “U” shape through the adjacent loops of the Akreos lens. With the serrated retina forceps positioned in the sclerotomy, the corresponding end of the suture was introduced into the anterior chamber using McPherson forceps, through the main incision, and then pulled through the sclerotomy. This movement was repeated successively at each of the four sclerotomies.

**Figure 2.**
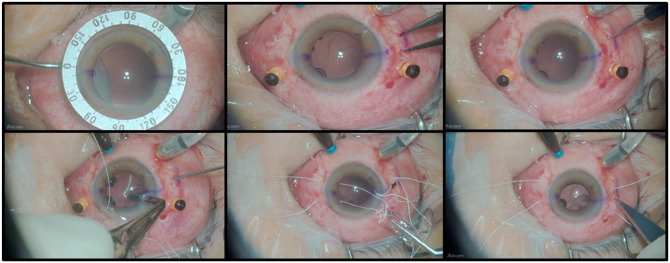
Surgical steps for fixation of the Akreos (Bausch + Lomb) intraocular lens (IOL) using CV-8 Gore-Tex (W.L. Gore & Associates) sutures. (A) Marking of 2 diametrically opposed scleral fixation points; (B) measurement and marking of the sclerotomies, spaced 4 mm apart; (C) creation of the inferotemporal sclerotomy using a 23-gauge trocar; (D) passage of the Gore-Tex suture through the sclerotomy; (E) positioning of the Akreos IOL with forceps at the intraocular implantation site; (F) creation of a scleral groove after final positioning of the IOL.

The main incision was enlarged with the 2.75-mm blade, and the IOL was folded along its longest axis with the assistance of the McPherson forceps. Subsequently, using the same forceps, the lens was positioned inside the eye. The sutures were pulled so that the lens was centralized. A scleral groove was made with a#11 blade between the adjacent sclerotomies, and their respective sutures were tied using the 3-1-1 technique. The knot of the suture was buried, and the conjunctiva was closed with an 8.0 Vicryl suture. The accessory and main incisions were hydrated with balanced saline solution, and in case of leakage, sutured with 10-0 nylon.^2,3^ The surgical steps of PPV with scleral fixation of the Akreos lens and Gore-Tex suture are shown in Figure 2.

### Clinical Evaluation

All participants were invited to a new in-person evaluation related to the current study. The protocol included intraocular pressure assessment using a Goldmann tonometer, subjective refraction, corneal topography with a Pentacam HR (Oculus, Optikgeräte GmbH, Wetzlar, Germany), and an optic disc cupping assessment by indirect ophthalmoscopy. All participants signed an informed consent document prior to study participation. Complications such as corneal edema, ocular hypertension, hypotony, hyphema, IOL decentration, cystoid macular edema, vitreous hemorrhage, suture breakage, retinal detachment, and uveitis-glaucoma-hyphema syndrome were noted. IOL decentration was evaluated by anterior segment biomicroscopy with a slitlamp. Ocular hypertension was defined as an intraocular pressure greater than or equal to 25 mm Hg. Any immediate complications that were documented in the medical records were also included in the analysis.

### Statistical Analysis

Statistical analyses were performed using Stata/SE Statistical Software (version 14.0, Stata Corp). Frequency tables were used for descriptive analysis. *χ*^2^ and Mann-Whitney tests were applied to compare, respectively, the categorical and continuous variables between groups. Multilevel mixed models were applied to investigate the effects of surgical technique on the outcomes, adjusting for sex, age, and follow-up time. For all tests, *P* values ≤ .05 were considered statistically significant. Mean values are ± SD.

## Results

A total of 20 eyes from 20 participants were selected for this study. Patients were classified by surgical technique and placed into 1 of 2 groups: Akreos plus GoreTex (n = 11) or MA60AC plus Prolene (n = 9). Table 1 presents the demographic characteristics of all participants.

**Table 1.** Demographic Data by Surgical Group.

	Akreos + Gore-Tex	MA60AC + Prolene	*P* Value
Sex, n (%)			.658
Male	6 (54.55)	5 (55.56)	
Female	5 (44.45)	4 (44.44)	
Age (y)			.357
Mean ± SD	61.36 ± 15.77	52.56±28.64	
Median	63.00	58.00	

There was no statistically significant difference between the groups in terms of sex (*P* = .658) or age (*P* = .357), allowing for a comparison of parameters, without age- or sex-related bias. The mean follow-up duration of the eyes included in this study was 1.65 ± 0.45 years (median, 1.63) in the Akreos group and 2.55 ± 1.43 years (median, 2.99) in the MA60AC group. This difference was not statistically significant (*P* = .172). Table 2 shows the postoperative results according to group, evaluated using multivariable mixed-effects models adjusted for sex, age, and follow-up duration.

**Table 2.** Postoperative Results by Surgery Type.

Parameter	Akreos + Gore-Tex	MA60AC + Prolene	*P* Value
Spherical equivalent (D)			.371
Mean ± SD	−5.10 ± 6.78	−2.96 ± 5.87	
Median	−3.37	−1.25	
Cylindrical component of refraction (D)			.934
Mean ± SD	−1.48 ± 1.63	−1.63 ± 1.38	
Median	−1.00	−2.00	
K1 (D)			.083
Mean ± SD	40.48 ± 2.33	42.16 ± 1.98	
Median	41.00	42.02	
K2 (D)			.910
Mean ± SD	44.86 ± 3.32	44.78 ± 2.10	
Median	45.00	45.05	
Corneal astigmatism (D)			.197
Mean ± SD	4.39 ± 3.48	2.61 ± 1.32	
Median	3.70	2.74	

Abbreviation: K1, K2, keratometry values.

The results reveal no difference between the 2 groups in terms of refractive outcomes (*P* > .05). Table 3 displays the results of the analyses of complications observed postoperatively.

**Table 3.** Complications According to Implant Type.

	Number (%)		
Complication	Akreos + Gore-Tex	MA60AC + Prolene	*P* Value
Corneal edema	4 (36.36)	2 (22.22)	.642
Ocular hypertension	3 (27.27)	0 (0.00)	.218
Hyphema	1 (9.09)	0 (0.00)	.550
IOL decentration	0 (0.00)	1 (11.11)	.450

Abbreviation: IOL, intraocular lens.

There was no statistically significant difference between the groups regarding the occurrence of corneal edema, ocular hypertension, hyphema, or IOL decentration (*P* > .05). No cases of hypotony, cystoid macular edema, vitreous hemorrhage, suture breakage, retinal detachment, or uveitis-glaucoma-hyphema syndrome were identified.

## Conclusions

The present study compared the outcomes of scleral fixation of IOLs using 2 different surgical approaches: Akreos lenses with Gore-Tex 8.0 sutures and MA60AC lenses with 10-0 Prolene sutures. The results showed no statistically significant differences between the 2 groups in terms of refractive outcomes, postoperative complications, or demographic variables, suggesting that both techniques have similar efficacy and safety profiles.

The refractive outcomes, including spherical equivalent, cylindrical component, and corneal astigmatism, did not significantly differ between the groups. This indicates that both the Akreos plus Gore-Tex and MA60AC plus Prolene approaches are equally effective in achieving the desired refractive goals. These findings also align with previous studies demonstrating that the choice of IOL type and suture material may not have a significant impact on refractive outcomes when the proper surgical technique is used.^1^

The incidence of postoperative complications, including corneal edema, ocular hypertension, hyphema, and IOL decentration, was not significantly different between the 2 groups. Corneal edema was the most frequent complication observed, occurring in 36.4% of eyes in the Akreos plus Gore-Tex group and 22.2% of eyes in the MA60AC + Prolene group. While these rates seem relatively high, all cases were transient and resolved with conservative treatment. In their recent meta-analysis, Kanclerz et al reported an overall transient corneal edema rate of 11.9% following scleral fixation, which is lower than that of our series but reflects a wide reported range depending on surgical technique, case complexity, and patient characteristics.

Notably, anterior chamber IOL placement showed the highest rate of corneal edema in that analysis (29.9%), highlighting the fact that postoperative edema is not uncommon in scenarios involving compromised ocular anatomy or complex interventions.^8^ Notably, no severe complications, such as suture breakage, retinal detachment, or uveitis-glaucoma-hyphema syndrome, were observed, reinforcing the safety of both surgical methods. The absence of IOL decentration in the Akreos plus Gore-Tex group may suggest that this method offers more reliable IOL stability, though the sample size was too small to confirm this with statistical significance.

Gore-Tex sutures have emerged as a strong alternative to Prolene owing to their durability and resistance to erosion over time. The similar outcomes observed in this study support the growing preference for Gore-Tex in scleral fixation procedures. Although not statistically significant, the trend toward fewer ocular hypertension cases in the Prolene group warrants further investigation in larger cohorts, as it may indicate a potential advantage in reducing postoperative pressure complications.^4^ Previous studies with Prolene sutures, however, have reported ocular hypertension as the primary early postoperative complication, reaching up to 30% of cases, similar to the results observed in our Gore-Tex group.^9–11^

The findings of this study have important implications for clinical practice, particularly in cases where scleral fixation of IOLs is necessary because of inadequate capsular support. The comparable outcomes between the 2 groups suggest that surgeons can choose between Akreos plus Gore-Tex and MA60AC plus Prolene based on availability, cost, or personal preference, without compromising patient outcomes.

This study has several important limitations, including its retrospective design, small sample size, and relatively short follow-up duration. Additionally, it was conducted at a single tertiary center, which may limit the generalizability of the findings to broader populations or different surgical settings. Given the limited number of cases, the results should be interpreted with caution and should not be used to draw definitive conclusions or make strong clinical recommendations. Instead, our findings contribute preliminary evidence that may inform future research. Studies with larger, multicenter cohorts and extended follow-up periods are needed to validate these observations and assess long-term safety and efficacy more robustly.

In conclusion, the study demonstrates that both Akreos plus Gore-Tex and MA60AC plus Prolene scleral fixation techniques are effective and safe for patients requiring IOL implantation in the absence of adequate capsular support. While the choice of suture material and lens type may not significantly impact refractive or safety outcomes, the durability of Gore-Tex sutures may offer a slight advantage in certain clinical scenarios. Further research is needed to solidify these observations and guide future clinical decisions.
